# Hydroxyurea Facilitates Manifestation of Disease Relevant Phenotypes in Patients-Derived IPSCs-Based Modeling of Late-Onset Parkinson’s Disease

**DOI:** 10.14336/AD.2018.1216

**Published:** 2019-10-01

**Authors:** Yuan Tan, Minjing Ke, Zhijian Huang, Cheong-Meng Chong, Xiaotong Cen, Jia-Hong Lu, Xiaoli Yao, Dajiang Qin, Huanxing Su

**Affiliations:** ^1^State Key Laboratory of Quality Research in Chinese Medicine, Institute of Chinese Medical Sciences, University of Macau, Macao, China.; ^2^South China Institute for Stem Cell Biology and Regenerative Medicine, Guangzhou Institutes of Biomedicine and Health, Chinese Academy of Sciences, Guangzhou, China.; ^3^Department of Neurology, National Key Clinical Department and Key Discipline of Neurology, First Affiliated Hospital, Sun Yat-Sen University, Guangzhou, China.

**Keywords:** disease phenotypes, ER stress, induced pluripotent stem cells, Parkinson’s disease, hydroxyurea

## Abstract

Induced pluripotent stem cells (iPSCs)-derived dopaminergic neurons might be reset back to the fetal state due to reprogramming. Thus, it is a compelling challenge to reliably and efficiently induce disease phenotypes of iPSCs-derived dopaminergic neurons to model late-onset Parkinson’s disease (PD). Here, we applied a small molecule, hydroxyurea (HU), to promote the manifestation of disease relevant phenotypes in iPSCs-based modeling of PD. We established two iPS cell lines derived from two sporadic PD patients. Both patients-iPSCs-derived dopaminergic neurons did not display PD relevant phenotypes after 6 weeks culture. HU treatment remarkably induced ER stress on patients-iPSCs-derived dopaminergic neurons. Moreover, HU treatment significantly reduced neurite outgrowth, decreased the expression of p-AKT and its downstream targets (p-4EBP1 and p-ULK1), and increased the expression level of cleaved-Caspase 3 in patients-iPSCs-derived dopaminergic neurons. The findings of the present study suggest that HU administration could be a convenient and reliable approach to induce disease relevant phenotypes in PD-iPSCs-based models, facilitating to study disease mechanisms and test drug effects.

Parkinson's disease (PD) is the second most common neurodegenerative disorder, with only Alzheimer's disease being more prevalent [1, 2]. Commonly used drugs, such as levodopa and other dopamine agonists, are only effective at the early stages of PD. They quickly become ineffective and even have severe adverse effects as the disease progresses [3]. Developing new pharmaceutical treatments for PD is urgently needed. Induced pluripotent stem cells (iPSCs) provide new opportunities for modeling PD to serve the drug screening platforms because of their abilities to proliferate indefinitely and differentiate into disease-relevant cell types [4-6]. The onset of PD generally occurs on patients over 50 years old. This suggests that aging is the strongest risk factor for the development of PD. Somatic cell reprogramming would reset the cell identity back to the embryonic state and result in the loss of particular age-associated features [6-8]. For example, cortical neurons derived from human iPSCs require several months, or even one year of continuous culturing to mature [9-13]. Many studies have reported that dopaminergic neurons derived from human iPSCs are relatively young and require several months of culturing to develop mature physiological features [14, 15]. This poses a great challenge for using iPSCs to model human late-onset diseases like PD.

When inducing disease phenotypes in iPSCs-derived lineages, it is common to expose the iPSC-derived cells to stressors, such as toxins and oxidants (16, 17). Recently another strategy was reported to manifest disease phenotypes by ectopic expression of progerin in induced fibroblasts and dopamine neurons derived from PD-iPSCs [6]. Previous studies have shown that progerin accumulation in the nuclear membrane destroys the function of lamin A and results in cell aging [18, 19]. The limitation of this technique is manipulating progerin expression in target cells is time-consuming and cost-ineffective. Therefore, developing an efficient and convenient approach to reliably induce disease phenotypes in PD-iPSCs-derived dopaminergic neurons is vitally desired for using iPSCs to model late-onset diseases.

Hydroxyurea (HU) was originally used as an anticancer agent, due to its ability to inhibit ribonucleotide reductase and block DNA synthesis [20, 21]. DNA synthesis disorders could disturb multiple processes that are involved in cell proliferation and metabolism. Previous studies have reported that HU could be used as a DNA replication inhibitor in order to induce senescence-like features in a number of mitotic cells, including K562, fibroblasts, and neural stem cells [21-24]. In the present study, we investigated if HU could be applied to induce disease phenotypes in post-mitotic neurons, such as dopaminergic neurons to model iPSCs-based late-onset diseases. Our study found HU treatment successfully induced endoplasmic reticulum (ER) stress in iPSC-derived dopaminergic neurons and promoted the manifestation of disease phenotypes in sporadic PD patients-derived iPSCs-based cell models.

## MATERIALS AND METHODS

### Fibroblasts culture and HU treatment

Fibroblasts were cultured in DMEM that was supplemented with 10% FBS, 100 units/ml penicillin, 100 µg/ml streptomycin, and 500 µg/ml L-glutamine at 37?°C with 5% CO2. The cells were seeded at 1.25 × 10^4^ cells per cm^2^ in 24-well plates (Eppendorf, Germany), treated with HU (Sigma, USA) at concentrations ranging from 1 mM, 8 mM and 16 mM for 4 days. The medium was replaced with fresh medium containing HU every 2 days.

### Relative cell number analysis

Fibroblasts were cultured at a density of 4 × 10^3^ cells/well in 96-well plates. The HU was dissolved in the fresh culture medium and was then added to the culture at the final concentration of 8 mM. The medium was replaced with fresh medium containing HU every 2 days. The cell number was measured by a Cell Counting Kit-8 (Beyotime Biotechnology) following the manufacturer’s guidelines. The percentage of the surviving cells after the HU treatment out of the total cells in the control group without HU treatment was presented.

### Senescence-associated-β-galactosidase assay

The cellular senescence was determined with senescence associated-β-galactosidase (SA-β-gal) staining, which was performed using a senescence associated-β-galactosidase staining kit (Cell Signaling Technology) in accordance with the manufacturer’s guidelines. The blue stained cells were evaluated from 10 different fields. The results were presented as a percentage of positive cells out of total cells.

### Establishment of PD patient-iPSCs

The generation of hiPSCs in the urine cells followed our previously reported protocol [25]. Urine cells were gathered from two donors with PD (with informed consent) based on IRB approval (no. GIBH-IRB02-2009002). The sequencing results did not reveal any mutations associated with PD. One patient carried a gene mutation (c.3671T>C, p.I1224T) in polymerase gamma (POLG) that encodes a highly conserved amino acid residue across multiple species. A total of ~500 ml of urine samples was collected mid-stream. Both donor samples were centrifuged to collect the exfoliated cells. The collected cells were cultured in a medium consisting of DMEM/F12 medium (Gibco) supplemented with 10% of FBS (Gibco), 0.1 mM non-essential amino acids (NEAA), 1 mM GlutaMAX (Life Technologies), 0.1 mM β-mercaptoethanol, and SingleQuot Kit CC-4127 REGM (Lonza).

When the urine cells were amplified to a sufficient quantity, an episomal pCEP4 vector that contained the miR302-367 precursor [26] and the other pCEP4 vector that carried OCT4, KLF4, SOX2, and SV40LT genes [27] were simultaneously transfected into the urine cells via nucleofection (Amaxa Basic Nucleofector Kit for primary mammalian epithelial cells, T-013 program, Lonza). The transfected urine cells were cultured in a Matrigel-coated 6-well plates (1-3×10^5^cells per well) with the urine cell culture medium for the first 2 days. The medium was changed to mTeSR1 and refreshed every 2 days for the remaining 13 days. Cell colonies were picked up and transferred to a new Matrigel-coated plate with mTeSR1 and 10 µM Y-27632. The culture medium was changed to a fresh mTeSR1 daily. The cells were dissociated to single cells for further cell expansion. The expressions of pluripotency genes Nanog and Oct4 were analyzed by real-time PCR and compared to the human embryonic stem cell line H9. Expressions of pluripotent markers Nanog and Oct4 were confirmed by immunofluorescence. The primary antibodies used were shown in Table S1. The proportion of iPSCs positive for human ES markers OCT4, SSEA4, or Tra-1-60 was quantified with Flow Cytometry (BD Biosciences). Karyotype analyses were performed to identify if the UC-iPSC cells had normal karyotype. In vivo pluripotency was evaluated with teratoma analyses. The iPS cell line derived from the patient with the POLG mutation was referred to as SPD-1 iPSCs and from the other sporadic patient sample was referred to as SPD-2-iPSCs.

### Dopaminergic neuron differentiation

Three iPS cell lines were induced to differentiate them into dopaminergic neurons. The SPD-1 iPSCs and the SPD-2 iPSCs were generated in this study. The UC-12-iPSCs were derived from a healthy donor [28]. Dopaminergic differentiation was performed as previously described, with some minor modifications [6, 14]. Briefly, iPSCs were disaggregated using Accutase for 2 min, centrifuged at 200×g for 3 min, plated on Matrigel-coated multiwells in the presence of Rock inhibitors at the density of 2×10^5^ cells/cm^2^, and then incubated with floor plate induction medium N1 containing SB431542 (10 μM; Tocris) and LDN193189 (100 nM; Miltenyi Biotec), SHH-C24 (100 ng/mL; Peprotech), FGF8 (100 ng/mL; Peprotech), and Purmorphamine (2 μM; Tocris) from day 0 to day 5. At day 3 until day 11, CHIR99021 (3 μM; Tocris) was added into the culture. N1 medium was gradually shifted to N2 medium starting on day 5 of differentiation, by mixing N1 and N2 in ratios of 75% (N1): 25% (N2) on day 5-6, 50% (N1): 50% (N2) on day 7-8, and 25% (N1):75% (N2) on day 9-10. At day 11, the cultures were split at a ratio of 1:1 using Accumax (STEM CELL) and cultured in N2 media supplemented with Y27632 (10 µM, SELLECKCHEM), LDN193189, CHIR99021. Finally, the cells were induced to the DA neuron fate with DA neuron differentiation medium. N1 medium (50 ml) contains 41 ml KO DMEM, 7.5 ml KO serum replacement, 0.5 ml Glutamax, 0.5 ml non-essential amino acids (NEAA), and 0.5 ml penicillin/streptomycin. N2 medium (50 ml) contains 48.5 ml DMEM/F12 with Hepes buffer/Neural basal, 0.5 ml N2 supplement, 0.5 ml Glutamax, and 0.5 ml penicillin/streptomycin. DA neuron differentiation medium (50 ml) contains 48 ml Neurobasal medium, 1 ml B27, 0.5 ml Glutamax, and 0.5 ml penicillin/ streptomycin. After 7 days in DA neuron differentiation medium, the cells were supplemented with 2% B27, 1% non-essential amino acids, 2 mM Glutamax, GDNF (PeproTech, 20 ng/mL), BDNF (PeproTech, 20 ng/mL), 0.2 mM ascorbic acid (Sigma Aldrich), DAPT (10 nM; Tocris), cAMP (10 μM, Sigma Aldrich), and TGFβ3 (1 ng/ml; R&D).

### Immunocytochemistry

Immunocytochemistry was performed to characterize the differentiated cells as described previously [29, 30]. The differentiated cells at each stage were fixed in 4 % paraformaldehyde (PFA) for 15 min at room temperature, washed with PBS, and then permeablized in PBS supplemented with 0.3 % Triton X-100, 10 % goat serum, and 3 % bovine serum albumin (BSA) for 1 h at room temperature. The cells were incubated with primary antibodies at 4 °C overnight, rinsed with PBS for 3 times, and stained with the appropriate Alexa Fluor-labeled secondary antibodies for 1 h at room temperature. The cells were counterstained with DAPI (Life Technologies) to visualize the nuclei. The images were taken with a Leica confocal microscope and analyzed with NIH ImageJ software. The primary antibodies used were shown in Table S1.

### Differentiated dopaminergic neurons treated with HU

After 10 days of dopaminergic induction, the cells were digested to single cells and seeded onto poly-L-ornithine/laminin-coated 24-well plates or 10 cm plates, at a density of 2-4 × 10^4^ cm^2^ at for another 10 days of culture in a dopaminergic maturation medium. They were then treated with 8 mM HU (Sigma, USA) for 4 days. The medium was replaced with a fresh dopaminergic maturation medium containing HU every 2 days.

### Neurite outgrowth assay

After treatment with 8 mM HU for 4 days, the neurons were fixed with 4% PFA and then stained with the anti-MAP2 antibody (Novex), followed with the Alexa Fluor 488 IgG secondary antibody (Life Technologies), and the DAPI (Sigma Aldrich). For measuring neurite length, images on the MAP2-positive neurons were obtained with an IN Cell Analyzer 2000 (GE Healthcare). Image analyses were conducted with NIH ImageJ software by an investigator who was blind to the experiment.

**Figure 1.**
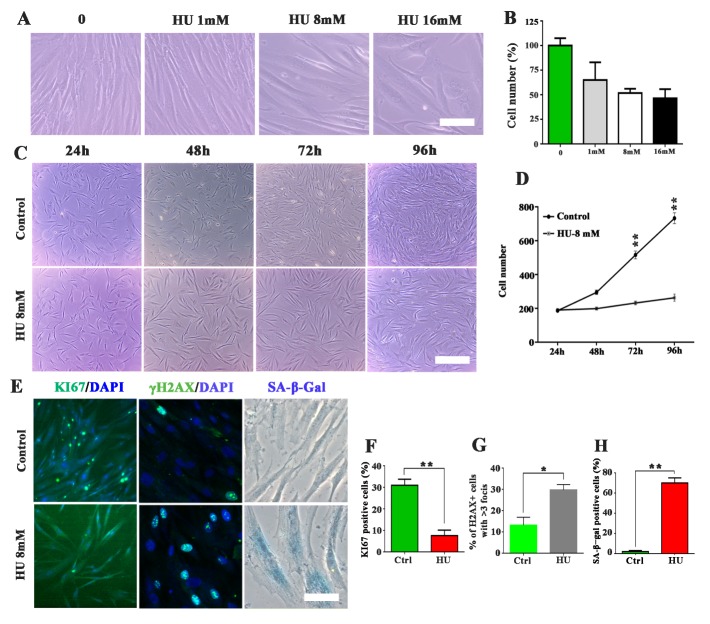
Hyroxyurea (HU) induced senescence-associated features in fibroblasts. (A) representative images of cell morphology and growth of human fibroblasts after treatment of HU at various concentrations for 4 days. (B) Cell number quantification after treatment of HU for 4 days. The cell number was represented by the percentage of survived cells after HU treatment compared to the total cells in the control group without HU treatment. The results were obtained from 3 independent experiments. (C) Representative images of the fibroblasts with 8 mM HU treatment for 4 days. (D) The number of cells in the visual field of phase contrast microscope. E: Representative images of the fibroblasts stained with Ki67, γH2AX, and SA-β-Gal. F-H: Quantification of positive staining and comparisons between the control group without HU treatment (Ctrl group) and the HU treatment group. The results were presented with averages ± SEM from 3 independent experiments, *p < 0.05, **p <0.01. Scale bar: 50 μm for A and E, 200 μm for C.

### Western Blot

The differentiated dopaminergic neurons with and without HU treatment were lysed using a RIPA buffer (Sigma Aldrich) supplemented with PMSF (Sigma Aldrich). The protein concentration was determined via the BCA. Equal amounts of protein were separated in SDS-PAGE gel and then transferred to a PVDF membrane. They were blocked in 5% BSA and then incubated in primary antibodies overnight followed by appropriate HRP-labeled secondary antibodies. Immunoreactive proteins were visualized with the BIO-RAD imaging system. The intensity of the protein band was determined with Image-Pro Plus analysis software. The primary antibodies used were shown in Table S1.

### Quantitative Real-time-PCR

Quantitative Real-time-PCR was performed to verify the expression level of genes that were related to pluripotency, dopaminergic lineages, and ER stress signal pathways. Total RNA was extracted from 3 iPS cell lines SPD-1 iPSCs, SPD-2 iPSCs, UC-12 iPSCs, and differentiated dopaminergic neurons with or without HU treatment using RNAzol®RT(MRC). The cDNA was synthesized with ReverTra Ace (TOYOBO) and Oligo (dT) 18 (TaKaRa). The reaction procedures took place at 42°C for 1h and 94°C for 5min. qRT-PCR was performed with SYBR® Premix Ex Taq (TaKaRa) using ViiA™ 7 Real-Time PCR System (Thermo). The reaction procedures began with an initial step at 95°C for 5 min, 40 cycles of 94°C for 15s, and then 60°C for 34s. The primers used were shown in Table S2.

### Gene Expression Analysis

Total RNA was extracted from the UC-12-iPSCs-derived dopaminergic neurons treated with the 8 mM HU for 4 days or without the treatment of HU using RNAzol®RT(MRC) from two independent experiments. The RNA-seq was analyzed by the Novogene Corporation. After quality control (QC) procedures of the total RNA, the mRNA was enriched using oligo (dT) beads, then fragmented randomly in a fragmentation buffer, and followed by cDNA synthesis using random hexamers and reverse transcriptase. After double-strand cDNA synthesis to generate the second strand by nick-translation, the cDNA library was generated with a set of purification, terminal repair, A-tailing, ligation of sequencing adapters, size selection, and PCR enrichment. Sequencing with HiSeq machines was performed after QC of cDNA library. The data analysis workflow included assessment of original data, mapping to referenced genome, expression quantification, and differential expression analysis such as transcription factors analysis, GO enrichment, protein-protein interaction analysis, and KEGG enrichment.

### Data Analysis

All experimental data were presented as the mean ± S.E.M. Analysis of variance (ANOVA) followed by post hoc Newman-Keuls multiple range tests was used for multiple comparisons. An unpaired t-test was used for the comparisons between two groups. For all analyses, the statistical significance was set to a *p* value <0.05. All analyses were performed using Prism (version 6.0; GraphPad, La Jolla, USA).

## RESULTS

### HU treatment induced senescence-associated markers in fibroblasts

The growth profiles of cultured human fibroblasts were investigated after they were treated in HU at various concentrations for 96 h. Treatment with concentrations of HU higher than 8 mM caused obviously abnormal cell morphology and may bring cell death (Fig. 1A). Cells in the culture treated with 8 mM HU ceased dividing but without obvious death (Fig. 1C and D). The 8 mM HU treatment reduced the mitotic capacity (Ki67), induced the DNA double-strand breaks (γH2AX), and increased senescence-associated β-galactosidase (SA-β-gal) staining activity in the cultured fibroblasts (Fig. 1E-H). Our observations indicated that 8 mM HU treatment successfully inhibited cell growth and induced the senescence-related phenotypes in fibroblasts.

### HU treatment induced ER stress in the UC-12-iPSCs-derived dopaminergic neurons

**Figure 2.**
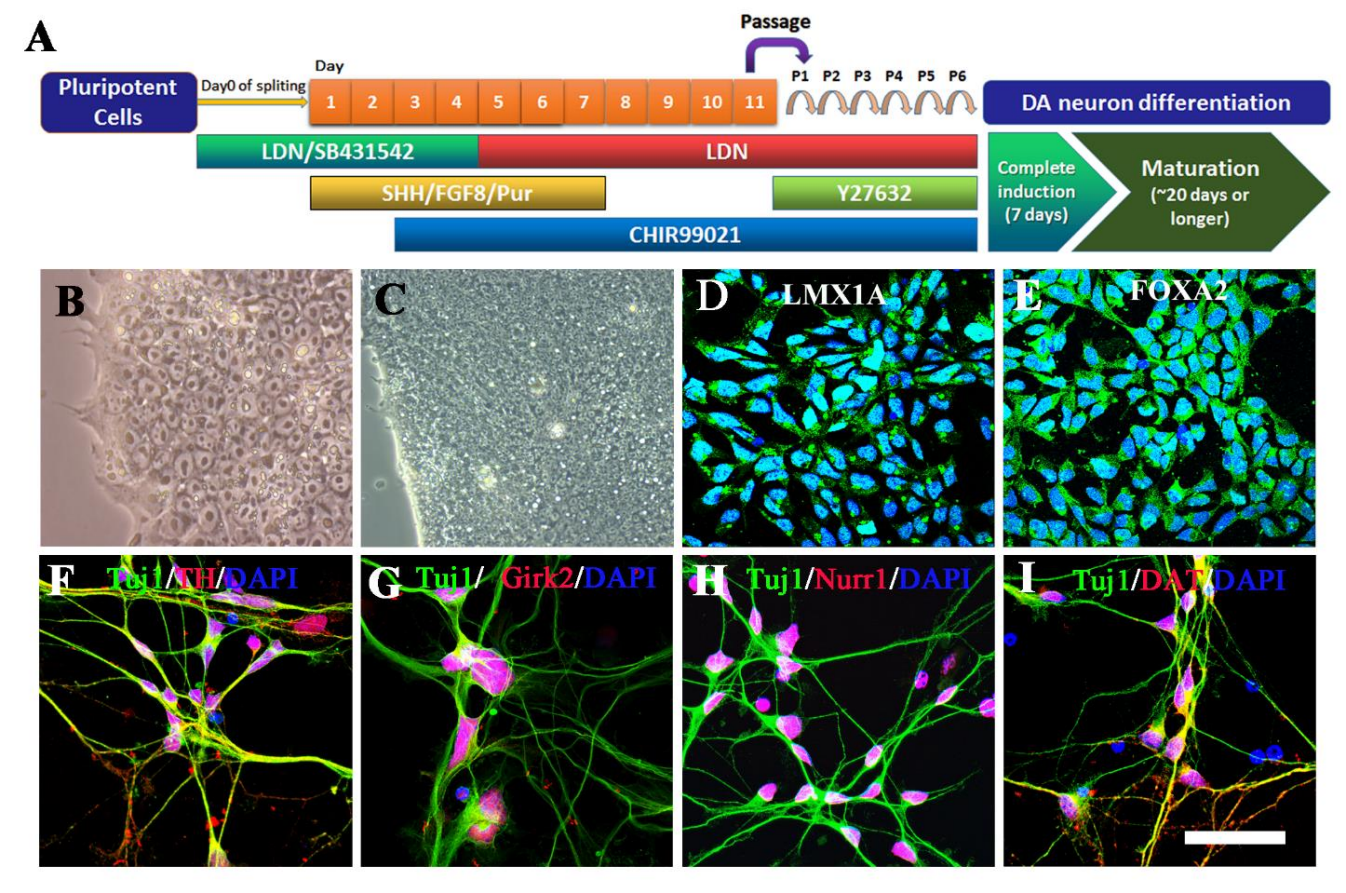
The dopaminergic differentiation of UC-12-iPSCs. (A) The workflow used dopaminergic differentiation of UC-12-iPSCs. (B) A representative phase contrast image of the UC-12-iPSCs. (C) A representative phase contrast image of UC-12-iPSCs under differentiation. (D) A representative image of LMX1A-positive midbrain floorplate precursors derived from the UC-12-iPSCs. E: A representative image of FOXA2-positive midbrain floorplate precursors derived from the UC-12-iPSCs. (F-I) Representative images of the dopaminergic neurons TH (F), Girk2 (G), Nurr1 (H), and DAT (I). Scale bar: 75 μm.

**Figure 3.**
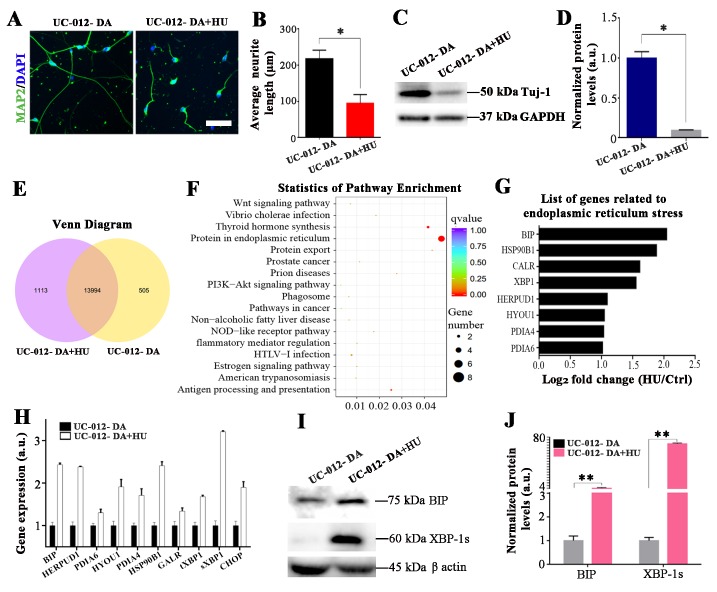
ER stress was induced by HU treatment in the UC-12-iPSCs-derived dopaminergic neurons. (A, B) The neurite length in the UC-12-iPSCs-derived dopaminergic neurons was dramatically reduced after 4 days of the HU treatment. The neurite was revealed by MAP2 staining. (C, D) Western blot analysis showed that the HU treatment decreased the expression of Tuj1 in UC-12-iPSCs-derived dopaminergic neurons. (E) The number of differentially expressed genes between the control group and the HU treatment group is shown with a Venn diagram. The middle circle indicated the number of mutual expressed genes between the control group and the HU treatment group. (F) The statistics of pathway enrichment analysis for the HU-induced aging in the UC-12-iPSCs-derived dopaminergic neurons. (G) Altered expression levels of genes related to the ER stress pathway. (H) The ER stress related genes were verified by qPCR. (I-J) The expression level of the key proteins in the ER stress pathways was further verified and quantified by western blot analysis. Scale bar: 45 μm for A and 120 μm for C.

We investigated if HU treatment could also inhibit cell growth and induce cellular senescence in human iPSC-derived dopaminergic neurons. The UC-12-iPSCs were derived from a healthy donor [28]. We performed dopaminergic differentiation following the well-established protocols with minor modifications [6, 14]. The experimental procedures for dopaminergic differentiation were shown in Fig. 2A. The UC-12-iPSCs were induced to differentiate into LMX1A-positive and FOXA2-positive midbrain floorplate precursors, an early stage of mDA neurons after 11 days of differentiation (Fig. 2B-E). These LMX1A-positive and FOXA2-positive midbrain floorplate precursors were fully matured after 7 days of complete dopaminergic induction and further around 20 days of maturation, as evidenced by expression of typical DA neuron markers such as TH (Fig. 2F), Girk2 (Fig. 2G), Nurr1 (Fig. 2H), and DAT (Fig. 2I). The mDA neurons were treated with 8 mM HU for 4 days. The HU treatment significantly shortened the dendrite length and decreased the protein expression of Tuj1 in the UC-12-iPSCs-derived dopaminergic neurons (Fig. 3A-D), suggesting that the growth of the UC-12-iPSCs-derived dopaminergic neurons was obviously inhibited. We analyzed the differentially expressed genes of the UC-12-iPSCs-derived dopaminergic neurons with and without HU treatment (Fig. 3E). The most differentially expressed genes were concentrated around the ER stress pathway (Fig. 3F). This suggested that HU inhibited the growth of the UC-12-iPSCs-derived dopaminergic neurons possibly acting through ER stress pathways. Further analysis revealed that the ER stress-related genes were upregulated in HU-treated iPSC-derived dopaminergic neurons (Fig. 3G). The elevated expression levels of the ER stress-related genes included BIP, tXBP1, sXBP1, and CHOP in the HU-treated iPSC-derived dopaminergic neurons. These were confirmed with the qPCR analysis (Fig. 3H). The western blot analysis further suggested that the two key ER stress proteins (BiP and sXBP1) were significantly elevated in the HU-treated dopaminergic neurons (Fig. 3I and J). These results showed that the HU treatment induced the ER stress of the UC-12-iPSCs-derived dopaminergic neurons.

### Two sporadic PD-iPSCs were established for study of disease phenotypes

Somatic cell reprogramming has been reported to reset the cell identity back to the embryonic state, resulting in the loss of particular senescence and age-associated features [5, 7, 8]. This could impede the PD iPSCs-based models when manifesting disease phenotypes. In the present study, two iPS cell lines generated from two sporadic PD patients, with informed consent. The sequencing results did not show any mutations that are implicated in the development of PD in these two PD patients. Both the SPD-1 iPSCs and the SPD-2 iPSCs expressed pluripotent markers such as OCT4, SSEA4, TRA-1-60, ZFP42, TERT, LIN28, and SOX2. These markers were revealed by immunostaining, flow cytometry, and qPCR analysis. Both the SPD-1 iPSCs and the SPD-2 iPSCs showed normal karyotypes (Supplementary Fig. 1 and 2).

**Figure 4.**
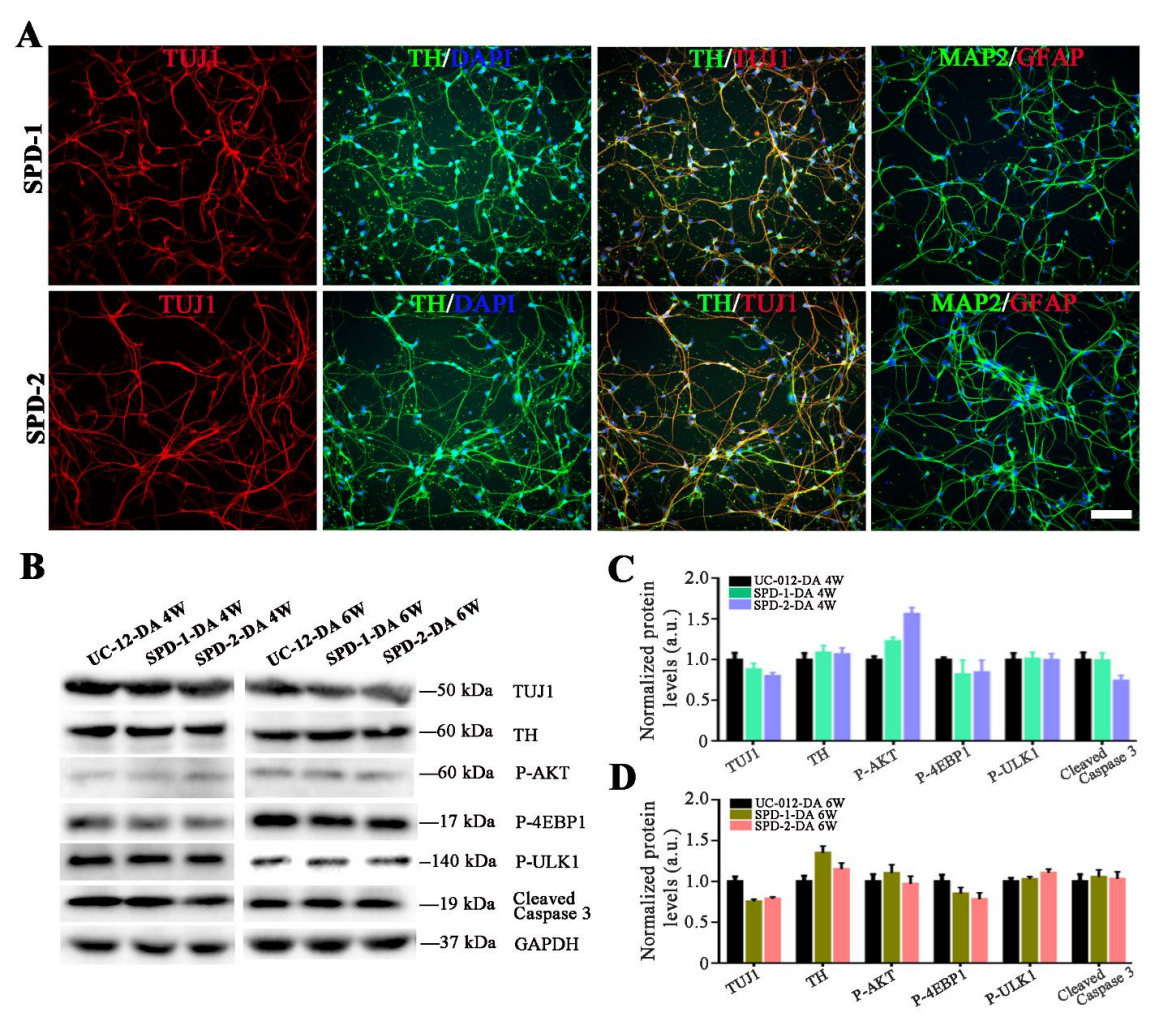
Dopaminergic neurons differentiated from the SPD-1 and the SPD-2 iPSCs. (A) Representative immunostaining images on differentiated neurons derived from the SPD-1 and the SPD-2 iPSCs. (B-D) The western blot analysis demonstrated that a lengthy culture did not enhanced the expression of key molecules related to PD-specific phenotypes in either the SPD-1 or the SPD-2 iPSCs-derived dopaminergic neurons. Scale bar: 80 μm.

We then induced these two PD iPS cell lines to differentiate into dopaminergic neurons. Both the SPD-1 and the SPD-2 iPSCs efficiently generated TH-positive neurons (Fig. 4A). Since these two iPS cell lines were derived from the PD patients, it was of interest to investigate if dopaminergic neurons derived from the PD-iPSCs could exhibit disease phenotypes to serve as cell models for PD. A set of biochemical markers including Tuj1, TH, p-AKT, its downstream signaling targets, and cleaved-Caspase 3 were used to analyze the cellular PD phenotypes. PD-iPSCs-derived dopaminergic neurons were cultured for either an additional 4 weeks or 6 weeks. Western blot analysis showed that both the SPD-1 and the SPD-2 iPSCs-derived TH-positive neurons did not display any molecular changes related to PD phenotypes, compared to the UC-12-iPSCs-derived dopaminergic neurons after the 4 weeks and 6 weeks culture (Fig. 4B-D).

### PD-iPSCs-based disease phenotypes were induced via HU treatment

**Figure 5.**
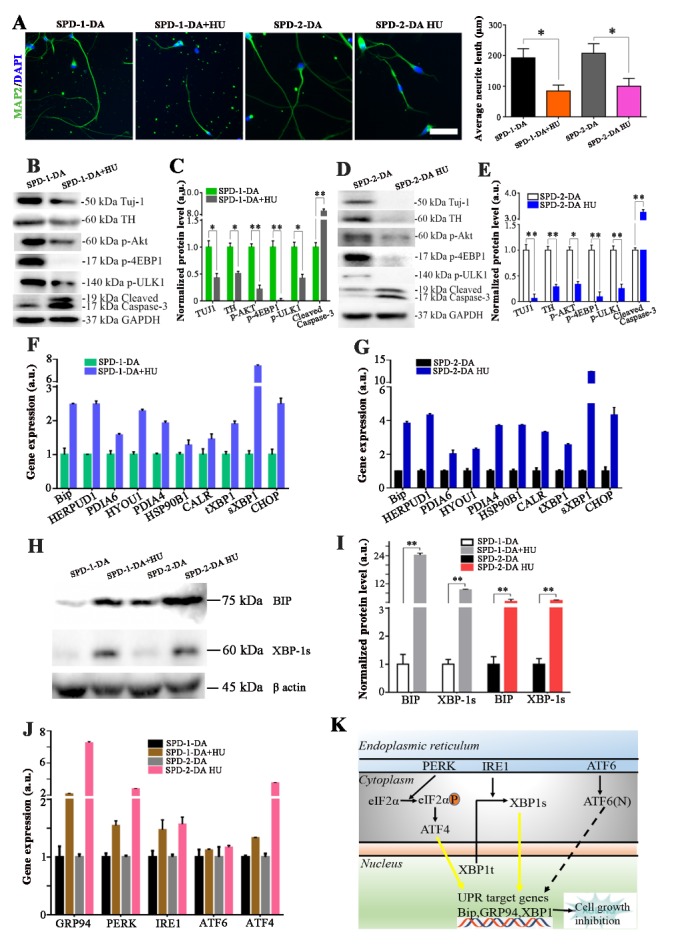
The PD-iPSCs-based disease phenotypes were induced via the HU treatment. (A) The HU treatment significantly reduced the neurite length in both the SPD-1 and the SPD-2 iPSCs-derived dopaminergic neurons. (B, C) Western blot analysis demonstrated that the HU treatment enhanced the expression level of the PD-associated proteins in the SPD-1 iPSCs-derived dopaminergic neurons. (D, E) Western blot analysis demonstrats that the HU treatment enhanced the expression level of the PD-associated proteins in SPD-2 iPSCs-derived dopaminergic neurons. (F, G) The ER stress related genes were verified by the qPCR in the HU-treated PD iPSC-derived dopaminergic neurons. (H, I) The expression level of the key proteins in the ER stress pathways in the HU-treated SPD-1 and the SPD-2 iPSCs-derived dopaminergic neurons were further verified and quantified by western blot analysis. J: The expression of the genes related to the ER stress pathways were further verified by the qPCR in the HU-treated PD iPSC-derived dopaminergic neurons. (K) Simplified scheme of the unfolded protein response (UPR) signaling pathway. Three different branches of the UPR were activated by ER stress: IRE1, PERK, and ATF6. The IRE1 spliced the cytosolic XBP1 mRNA to obtain the XBP1s transcription factor, which helped to resolve the ER stress [34, 35]. The PERK initiated the UPR by phosphorylation of eIF2a, which attenuated the global protein synthesis and contributed to restoring the ER homeostasis [36]. Yellow highlighted arrows indicate the two branches of the UPR signaling pathways that could take part in HU-induced cellular senescence. Scale bar: 80 μm.

No reliable disease phenotypes were exhibited in the SPD-1 and the SPD-2 iPSCs-derived dopaminergic neurons after 4 weeks or 6 weeks culture. Several months-culture could be required to induce disease phenotypes for SPD-iPSCs-based disease models. Prolonged culture is time consuming and would lead to many uncertainties regarding the analysis of cultured iPSCs-derived neurons. We treated UC-12, SPD-1, and SPD-2 iPSCs-derived TH-positive neurons with 8 mM HU for 4 days. We first investigated whether HU could induce the senescence-related phenotypes in these iPSCs-derived dopaminergic neurons. Nuclear morphology abnormalities and loss of heterochromatin markers are known biomarkers for cellular senescence. Immunostaining with LAM A/C and H3K9me3 showed no disintegration of the nuclear organization and the decrease of the heterochromatin marker expression, suggesting that no senescent phenotypes were detected in UC-12, SPD-1, and SPD-2 iPSCs-derived dopaminergic neurons after HU treatment (Supplementary Fig. 3). We then investigated if the HU could facilitate PD iPSC-derived dopaminergic neurons to manifest PD-associated disease phenotypes. Both the SPD-1 and the SPD-2 iPSCs-derived TH-positive neurons exhibited significantly shortened neurites after the HU treatment (Fig. 5A). Western blot analysis showed that the expression of Tuj1 and TH protein significantly decreased in the SPD-1 and the SPD-2 iPSCs-derived TH-positive neurons with HU treatment, when compared to SPD-1 and the SPD-2 iPSCs-derived TH-positive neurons without HU treatment (Fig. 5B-E). Moreover, HU treatment decreased the expression of p-AKT and its downstream targets (p-4EBP1 and p-ULK1) and increased the expression level of cleaved-Caspase 3 in both the SPD-1 and the SPD-2 iPSCs-derived TH-positive neurons (Fig. 5B-E). Interestingly, the expression of Tuj1, TH, and p-AKT was decreased in UC-12 iPSCs-derived dopaminergic neurons after HU treatment, but no change in the expression level of cleaved-Caspase 3 was found within 6 weeks culture (Supplementary Fig. 4). These results demonstrated that HU treatment successfully induced the SPD-iPSCs-based disease phenotypes as evidenced by the morphological and the biochemical changes of in SPD-iPSCs-derived dopaminergic neurons.

To our expectation, the expression level of the ER stress-related genes, including BIP, tXBP1, sXBP1, and CHOP was significantly upregulated in both the SPD-1 and the SPD-2 iPSCs-derived TH-positive neurons after the HU treatment (Fig. 5F and G). The expression of two key ER stress related proteins (BiP and sXBP1) were significantly elevated in the PD iPSC-derived TH-positive neurons after HU treatment (Fig. 5H and I). The ER stress response is mediated by 3 types of ER stress sensors: IRE1a, PERK, and ATF6. The real-time PCR analysis revealed that HU treatment activated the expressions of GRP94, IRE1a, PERK, and ATF4, but did not change the expression of ATF6 in both SPD-1 and the SPD-2 iPSCs-derived TH-positive neurons (Fig. 5J). We suggested that the HU treatment acts on the PERK and the IRE1a pathways, and subsequently leads to ER stress and UPR dysfunction which trigger cell death by apoptosis (Fig. 5K).

## DISCUSSION

Using patients-derived iPSCs to model PD offers a remarkable opportunity for disease mechanistic study and drug discovery. The ability to appropriately induce disease phenotypes *in vitro* is critical for successfully establishing iPSCs-based late-onset diseases like PD. The present study showed that HU treatment facilitated the manifestations of PD phenotypes in two SPD patients-derived iPSCs models.

No effective drugs are currently available for the treatment of PD. There is a lack of understanding of the underlying mechanisms for PD development, which represents a critical barrier when developing effective therapies. Using PD patients-derived iPSCs-based models to recapitulate the disease is a powerful tool to explore the mechanisms involved in the progression of PD and test disease-targeted therapies. PD patients-derived iPSCs-based models alone are unable to express the clinical symptoms, such as rigidity, bradykinesia, and tremor. Therefore, key molecular and cellular changes involved in the disease development are widely used to recapitulate PD-related phenotypes *in vitro*. A set of molecular hallmarks, including Tuj1, TH, p-AKT, its downstream signaling targets, cleaved-Caspase 3, and reduced neurite outgrowth are used to analyze the PD cellular phenotypes in the present study. Tuj1 is a general neuronal marker and decline of Tuj1 expression indicates the neuronal loss. TH catalyzes the formation of L-DOPA, which is the rate-limiting step in the synthesis of dopamine and decline of TH expression results in lowered dopamine synthesis, which directly leads to PD. Decreased levels of p-AKT and its downstream signaling targets such as p-4EBP1 and p-ULK could cause neuronal death in PD [31-33]. Cleaved-Caspase 3 is a sensitive marker for cell apoptosis. These molecular and cellular changes illustrate the specific disease-related phenotypes such as neurite degeneration, mitochondrial dysfunctions, and neuronal degeneration. It is noted that more molecular and cellular functional changes could be used to recapitulate PD-related phenotypes *in vitro*. For example, α-synuclein is one of the typical pathological hallmarks for many genetic and sporadic cases [34]. In this study, we did not detect any α-synuclein accumulation in those SPD iPSCs-derived TH-positive neurons, even with the HU treatment. We proposed that α-synuclein accumulation *in vitro* possibly needs long-term culture. Previous research also suggested that autophagy was impaired in iPSCs-derived dopaminergic neurons isolated from idiopathic and LRR2-mutant PD patients [35]. Therefore, we need to study whether autophagy impairment can be found in those PD iPSCs-derived TH-positive neurons and whether HU treatment could accelerate autophagy impairment in PD iPSCs-derived dopaminergic neurons in our future studies.

Aging is the strongest risk factor for many neurodegenerative diseases [36-38]. There are many factors associated with cell aging including cellular senescence [39]. Developing strategies that would artificially induce cellular senescence and aging in iPSCs-derived neuronal subtypes is integral for modeling late-onset diseases. Miller and colleagues utilized a transient expression of progerin in human iPSCs-derived dopaminergic neurons and successfully induced an age-dependent neurodegeneration of PD [6]. This study demonstrates that progerin-induced aging is an effective approach to modeling late-onset diseases. However, there are some concerns related to the use of progerin-induced aging, which include the laborious preparation of modified-RNA of progerin and the efficiency of the transfection of dopaminergic neurons to induce progerin expression. HU was initially utilized as an anticancer drug, due to its ability to repress the ribonucleotide reductase and decrease the production of the deoxynucleotides required for DNA synthesis [40]. Previous studies have shown that HU induces senescence-like features in a number of mitotic cells, including K562, fibroblasts, and neural stem cells [21-24]. We did not detect any senescent makers in iPSCs-derived dopaminergic neurons after HU treatment, suggesting that HU might be unable to induce cellular senescence in post-mitotic cells. The exact mechanisms for cellular senescence in terminally differentiated cells as dopaminergic neurons contributing to ageing and age-related disease is unclear [41, 42]. One of our hypotheses is that mitotic cells are more vulnerable to HU treatment and easier to exhibit senescence related phenotypes compared to post-mitotic cells. Since post-mitotic cells such as dopaminergic neurons do not need to vitally synthesize DNA, they possibly need much longer time to express senescence-like markers after HU treatment. It is really necessary to investigate whether HU could induce senescence on human iPSCs-derived dopaminergic neurons in a long-term culture window beyond 6 weeks.

Our findings demonstrate that HU treatment activates the ER stress pathways in iPSCs-derived dopaminergic neurons. Interestingly, ER stress is one of the pathogenic mechanisms for the development of PD [43, 44]. There are many other pathogenic mechanisms for PD including α-Synuclein accumulation [45], oxidative injury [46], mitochondrial dysfunction [47], autophagy dysfunction [48], and etc. Indeed, ER stress is a common feature of most neurodegenerative diseases. Recent evidence suggests that ER stress acts as a driver of brain aging [49]. The results of the present study suggest that HU treatment induced PD-related phenotypes mainly via ER stress pathway. But future studies are absolutely needed to address the other mechanisms by which HU promotes disease phenotypes in patients-derived iPSCs-based models.

In conclusion, the etiology of sporadic PD is not well defined and commonly considered an interaction between environmental factors and a number of genetic variants. There is a need for more studies using PD-iPSCs-based models to explore underlying disease mechanisms. The findings of the present study suggest that HU could be applied to induce disease phenotypes in sporadic PD-iPSCs-based models to gain mechanistic insights. This would contribute to mechanistic studies and high throughput screening for drug candidates for the treatment of PD.

## Supplementary Materials

The Supplemenantry data can be found online at: www.aginganddisease.org/EN/10.14336/AD.2018.1216
